# Gestational hypertension as a factor associated with chronic kidney disease: the importance of obstetric history of women undergoing hemodialysis

**DOI:** 10.1590/2175-8239-JBN-2022-0119en

**Published:** 2023-01-09

**Authors:** Beatriz Tenorio Batista Carvalho, Anderson Borovac-Pinheiro, Sirlei Siani Morais, José Paulo Guida, Fernanda Garanhani Surita

**Affiliations:** 1 Universidade Estadual de Campinas, Faculdade de Ciências Médicas, Departamento de Ginecologia e Obstetrícia, Campinas, SP, Brazil.

**Keywords:** Reproductive Histor, Renal Dialysi, Pregnancy Complication, Kidney Failur, Chroni, Pré-Eclâmpsi, Hypertension, História Reprodutiva, Diálise Renal, Complicações na Gravidez, Falência Renal Crônica, Pre-Eclampsia, Hipertensão

## Abstract

**Introduction:**

Pregnancy-related complications may impact women’s reproductive cycle and health through their lives. The objective of this study was to evaluate the sociodemographic, clinical, and obstetric history of women undergoing hemodialysis.

**Methods:**

We performed a cross-sectional study in a specialized health facility with four hemodialysis units. Sociodemographic characteristics, clinical and personal history, obstetric and perinatal results of women with pregnancies before hemodialysis were evaluated. Prevalence, bivariate, and logistic regression analyses were performed.

**Results:**

We included 208 (87.76%) women. Hypertension was the main cause of chronic kidney disease (CKD) (128 women). Rates of adverse perinatal outcomes, including prematurity, low birth weight, miscarriage, fetal death, and neonatal death, were 19.3%, 14.5%, 25.5%, 12.1%, and 5.3%, respectively. Hypertensive syndromes during pregnancy occurred in 37.0% of women, with 12.5% reporting preeclampsia and 1.4% reporting eclampsia. Up to 1 year after birth, 45.2% of women reported hypertension. Hemodialysis due to hypertension was associated with a history of hypertension during pregnancy (OR 2.33, CI 1.27 – 4.24), gestational hypertension (2.41, CI 3.30 – 4.45), and hypertension up to one year after birth (OR 1.98, CI 1.11 – 3.51). Logistic regression showed that gestational hypertension was independently associated with CKD due to hypertension (aOR 2.76, CI 1.45 – 5.24).

**Conclusion:**

Women undergoing hemodialysis due to hypertension were more likely to have gestational hypertension or hypertension up to one year after birth. To delay end-stage renal disease, it is necessary to identify women at risk of kidney failure according to their reproductive history.

## RESUMO

**Introdução:**

Complicações relacionadas à gestação podem afetar o ciclo reprodutivo e a saúde das mulheres ao longo de suas vidas. Este estudo visou avaliar histórico sociodemográfico, clínico e obstétrico de mulheres em hemodiálise.

**Métodos:**

Realizamos estudo transversal em unidade de saúde especializada com quatro unidades de hemodiálise. Avaliou-se características sociodemográficas, histórico clínico e pessoal, resultados obstétricos e perinatais de mulheres com gestações anteriores à hemodiálise. Foram realizadas análises de prevalência, bivariadas e regressão logística.

**Resultados:**

Incluímos 208 (87,76%) mulheres. Hipertensão foi a principal causa de doença renal crônica (DRC) (128 mulheres). Taxas de desfechos perinatais adversos, incluindo prematuridade, baixo peso ao nascer, aborto espontâneo, óbito fetal e neonatal, foram de 19,3%, 14,5%, 25,5%, 12,1% e 5,3%, respectivamente. Síndromes hipertensivas durante a gestação ocorreram em 37,0% das mulheres, com 12,5% relatando pré-eclâmpsia e 1,4% relatando eclampsia. Até 1 ano após o parto, 45,2% das mulheres relataram hipertensão. Hemodiálise devido à hipertensão foi associada ao histórico de hipertensão na gestação (OR 2,33; IC 1,27 - 4,24), hipertensão gestacional (2,41; IC 3,30 - 4,45), e hipertensão até um ano após o parto (OR 1,98; IC 1,11 - 3,51). A regressão logística mostrou que hipertensão gestacional foi independentemente associada à DRC devido à hipertensão (ORa 2,76; IC 1,45 - 5,24).

**Conclusão:**

Mulheres submetidas à hemodiálise por hipertensão foram mais propensas a apresentar hipertensão gestacional ou hipertensão até um ano após o parto. Para retardar a doença renal em estágio terminal, deve-se identificar mulheres em risco de insuficiência renal de acordo com sua história reprodutiva.

## Introduction

Chronic kidney disease (CKD) is responsible for significant morbidity and mortality worldwide, with increasing incidence globally. In 2017, 697.5 million cases of CKD were recorded, with an overall prevalence of 9.1%. The overall rate of CKD mortality increased 41.5% between 1990 and 2017, culminating in 1.2 million deaths from CKD in 2017^1^. In Brazil, the prevalence of CKD is 1.4%, and it is estimated that there are currently 15 million patients, most of whom are not receiving treatment^2^.

CKD is a noncommunicable disease consisting of multiple heterogeneous structural and functional renal conditions with several causes and prognostic factors. Among the causes of CKD are glomerular diseases, diabetes mellitus, chronic hypertension, obesity, and smoking^1^.

CKD is a determinant risk factos for cardiovascular disease, accounting for 30% of deaths worldwide^3^. CKD is usually asymptomatic in the initial stages; however, end-stage renal disease (ESRD) requires renal replacement therapy or transplantation^4^. The distribution of CKD is similar in men and women; however, in women, CKD impacts reproductive function^5^.

In women, ESRD causes dysfunction of the hypothalamic-pituitary axis, with a consequent reduction in fertility. Amenorrhea and menstrual irregularity are common. It also increases the risk of unfavorable maternal and perinatal outcomes, including abortion, fetal growth restriction, preterm birth, hypertensive disorders, and infections^5^.

Many women undergoing hemodialysis do not have a known cause for CKD. There is evidence of an association between some adverse obstetric outcomes, such as preeclampsia, and the risk of CKD^6,7^. Hemodynamic and structural changes occur in the kidneys and segments of the urinary tract also occur in a normal high-risk pregnancy, so CKD can begin or worsen due to kidney overload imposed by a pregnancy^4^. Our work aimed to explore the reproductive history of women undergoing hemodialysis and to understand the impact of adverse perinatal outcomes (APO) as a factor associated with CKD due to hypertension.

## Methods

We performed a cross-sectional study to evaluate women undergoing hemodialysis in Campinas, a city in Southern Sao Paulo. Our data collection occurred in a specialized health facility comprising four hemodialysis units from August to December 2019. These units treat approximately 400 women undergoing at least three weekly sessions.

The selection of participants was intentional; we included women with a previous diagnosis of ESRD undergoing hemodialysis at any unit after agreeing to participate and signing an informed consent. We excluded women who had neurological or psychiatric diseases. We also excluded women with hearing diseases because they could not answer the questions.

We obtained data through face-to-face interviews performed by researchers and trained assistants using questionnaires created especially for this study. We evaluated sociodemographic characteristics, years on hemodialysis, personal history, obstetric and perinatal outcomes, and comorbidities. We obtained rates of comorbidities and ESRD cause in three gestational periods (pre-gestational, during pregnancy, and up to one year after birth). A specific database was created for this study in Microsoft Excel. The researcher conducted a quality-control assessment of data collection before and during the electronic entry of data into the database to identify possible inconsistencies.

In this analysis, we included women who had at least one pregnancy before the onset of hemodialysis. We excluded women with pregnancies after starting hemodialysis because we aimed to evaluate the impact of obstetric history as a factor associated with CKD.

The mean and standard deviation were used to describe continuous variables, and qualitative variables were described as frequency and percentage. We compared hypertension as a cause of CKD using the chi-square test, Fisher’s exact test, and Mann-Whitney test. We also obtained the odds ratio (OR) and confidence interval (CI) for this comparison. We performed a logistic regression using the stepwise criterion, including in the model all variables with a p-value below 0.25. The significance level was 5%, and the software used for statistical analysis was SAS, version 9.4.

We grouped all maternal adverse outcomes (gestational hypertension, preeclampsia, diabetes, hemorrhage, placenta abruption, eclampsia, preterm delivery) and all adverse fetal outcomes (fetal death, birth weight lower than 2500g, and neonatal death). We also built the variable “any perinatal adverse outcome” grouping the two previous variables.

We followed all items of the Strengthening the Reporting of Observational Studies in Epidemiology (STROBE) consensus for the writing of this manuscript (9).

The Institutional Review Board from the State University of Campinas, Brazil, approved the study (CAAE report: 15429419.5.0000.5404). The principles set out in Resolution 466/2012 (of 12/12/2012) of the National Health Council were followed. All participants signed an informed consent form.

## Results

During our data collection (August to December 2019), we interviewed 237 women; 11 (4.64%) became pregnant while on hemodialysis and 18 (7.60%) had no previous pregnancy. We included in the analysis 208 (87.76%) women with hemodialysis before pregnancy. Among those, 128 (61.54%) had hypertension as the cause of CKD, while 80 (38.46%) had other causes for CKD. These groups were further compared.

Table 1 shows the sociodemographic characteristics and morbidities of all included women. Their average age was 57.82 (±12.87) years, and 134 (64.42%) were on hemodialysis for less than five years. The interval between the last pregnancy and the onset of ESRD was greater than ten years for 100 (48.07%) women, while it was less than two years in 12 (5.77%). Hypertension was the most prevalent cause of ESRD (61.5%), followed by diabetes (30.8%). ESRD cause was unknown for 17 women (8.2%).

**Table 1.** Sociodemographic characteristics, habits, and morbidities among women on hemodialysis with at least 1 pregnancy

	n = 208	%
**Age**		
<50y	49	23.6
≥50y	159	76.4
**Marital status**		
Single	27	13.0
Stable relationship	103	49.5
Widow	44	21.2
Divorced	34	16.3
Profession*		
Paid work	110	53.1
Unpaid work	61	29.5
Retired	36	17.4
**Schooling**		
None	24	11.5
Fundamental	131	63.0
Middle school	49	23.6
College	4	1.9
**Area of living**		
Rural	10	4.8
Urban	198	95.2
**Skin color**		
White	79	38.0
Non-White	129	62.0
**Smoking**	48	23.0
**Alcohol**	19	9.1
Drugs*	5	2.4
**HIV positive**	2	1.0
**Hepatitis C**	8	3.8
**Hepatitis B**	4	1.9
**Renal failure cause #**		
Hypertension	128	61.5
Diabetes	64	30.8
Infectious	10	4.8
Unknown	17	8.2
Other	45	21.6
Years between last pregnancy and ESRD**		
≤2	12	6.0
3–10	41	20.6
>10	100	73.4
Hemodialysis in years***		
≤1	53	25.9
2–5	81	39.5
6–10	41	20.0
>10	30	14.6

*1 missed; **9 missed; ***3 missed; # can be more than one cause.

As shown in Table 2, the majority (144, 69.2%) of the included women had three or more previous pregnancies, and the age of the first pregnancy occurred before 20 years for 110 (53.1%) women. Regarding perinatal outcomes, 40 women (19.3%) reported preterm birth, 30 women (14.5%) had babies with low birth weight, 25 (12.1%) presented fetal death, 11 (5.3%) reported neonatal death, and 62 (29.8%) had any gestational loss.

**Table 2.** Gestational history and comorbidities before and up to 1 year after pregnancy among women on hemodialysis with at least 1 pregnancy

	n = 208	%
**Pregnancies**		
1-2	64	30.8
≥3	144	69.2
**Parity**		
0	1	0.5
1-2	76	36.5
≥3	131	63.0
**Miscarriages**		
0	155	74.5
≥1	53	25.5
**Living children**		
0	5	2.4
1-2	81	38.9
≥3	122	58.7
Age at first pregnancy*		
<20y	110	53.1
≥20y	97	46.9
Age at last pregnancy**		
<20y	9	4.8
20–29y	77	41.4
≥30y	100	53.8
**Morbidities before pregnancy**		
Hypertension	45	21.6
Urinary infection	39	18.8
Diabetes	17	8.2
Cardiopathy	4	1.9
Others	3	1.5
**Complications during pregnanc**y		
Hypertension	77	37.0
Preeclampsia	26	12.5
Diabetes	23	11.1
Hemorrhage	21	10.1
Placenta abruption	5	2.4
Eclampsia	3	1.4
**Perinatal outcome**		
Preterm birth	40	19.3
Low birth weight	30	14.5
Fetal death	25	12.1
Neonatal death	11	5.3
Gestational loss^#^	62	29.8%
Adverse perinatal outcome^##^	88	42.3% (Continue)
**Morbidities up to one year after birth**		
Diabetes	34	16.3
Hypertension	94	45.2
Cardiopathy	12	5.8
Urinary infection	32	15.4
Other	4	1.9

*1 missing value; **22missing values; ^#^Gestational loss: miscarriage + fetal and neonatal death; ^##^Adverse perinatal outcome: miscarriage + fetal and neonatal death + preterm birth + low birth weight.

**Figure 1.**
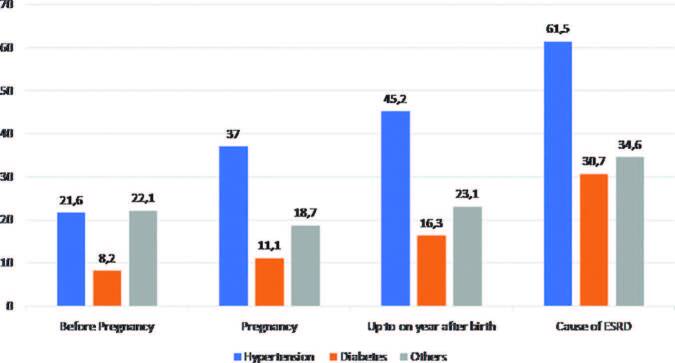
Percentage of primary comorbidities before, during, and up to one year after birth and cause of end-stage renal disease (ESRD) in women on hemodialysis.

Forty-five (21.6%) women reported hypertension before pregnancy, 77 (37.0%) reported hypertension during pregnancy, and 94 (45.2%) remained with hypertension up to one year after birth. Diabetes affected 17 (8.2%) women before pregnancy, 23 (11.1%) during pregnancy, and 34 (16.3%) after childbirth. Other causes of CKD included kidney infection and obstructive factors. Figure 1 illustrates the frequency of these conditions in the three periods and the cause of CKD.

Women on hemodialysis due to hypertension were more likely to have a history of any hypertensive syndrome during pregnancy (OR 2.33, CI 1.27 – 4.24) or gestational hypertension (OR 2.41, CI 1.30 – 4.45). Up to one year after birth, women under hemodialysis were more likely to present any hypertensive syndrome (OR 1.98, CI 1.11 – 3.51). Other comparisons are presented in Table 3.

**Table 3.** Bivariate analysis of sociodemographic characteristics, habits, obstetric history, and morbidities according to adverse perinatal outcomes among women on hemodialysis with at least 1 pregnancy

	Hypertension as cause of CKD (n = 208)					
	Yes (n = 128)		No (n = 80)		p-value#	OR (IC 95%)
	n	%	n	%		
**Pregnancies**					0.618	
1-2	41	32.0	23	28.7		Ref
≥3	87	68.0	57	71.2		0.86 (0.44 – 1.64)
**Usual miscarriage (2 or more)**	15	11.7	5	6.2	0.193	1.99 (0.69 – 5.71)
**First pregnancy before 15 years**	12	9.4	5	6.3	0.438	1.15 (0.83 – 1.60)
**Complications during pregnancy**						
Any hypertension	60	46.9	22	27.5	<**0.01**	**2.33 (1.27 – 4.24)**
Gestational hypertension	57	44.5	20	25.0	<**0.01**	**2.41 (1.30 – 4.45)**
Preeclampsia	17	13.3	9	11.2	0.67	1.21 (0.51 – 2.86)
Diabetes	14	10.9	9	11.2	0.94	0.97 (0.40 – 2.35)
Hemorrhage	14	10.9	7	8.7	0.61	1.28 (0.49 – 3.32)
Placenta abruption	5	3.9	0	0	0.07	NS
Eclampsia	2	1.6	1	1.3	0.86	1.24 (0.11 – 13.88)
Preterm delivery	20	15.6	20	25.0	0.09	0.55 (0.27 – 1.11)
Any maternal adverse outcome	75	58.6	36	45.0	0.06	1.73 (0.98 – 3.04)
Fetal Death	14	11.0	11	13.7	0.56	0.78 (0.33 – 1.81)
Birth weight <2500g	16	12.6	14	17.5	0.33	0.68 (0.31 – 1.48)
Neonatal death	6	4.7	5	6.2	0.63	0.74 (0.22 – 2.52)
Any adverse fetal outcome	29	22.8	23	28.7	0.34	0.73 (0.39 – 1.39)
Any perinatal adverse outcome	81	63.9	43	53.7	0.15	1.51 (0.85 – 2.68)
**Morbidities up to one year after birth**						
Any hypertension	66	51.6	28	35.0	**0.02**	**1.98 (1.11 – 3.51)**

Missing *1 **3 ***22 **** 9; ^#^Fisher’s exact test/Chi-square test.

In logistic regression, the following variables were included: recurrent abortion (two or more), gestational hypertension, placental abruption, preterm birth, any maternal adverse outcome, any perinatal outcome, and chronic hypertension. Gestational hypertension was independently associated with CKD due to hypertension (adjusted OR 2.76, CI 1.45 – 5.24, p-value < 0.01), while preterm birth was a protective factor (adjusted OR 0.44, CI 0.21 – 0.92, p-value = 0.03).

## Discussion

In this study, we aimed to understand the obstetric history of women undergoing hemodialysis due to CKD. In 208 women, hypertension was the leading cause of CKD, and it was associated with occurrence of gestational hypertension and hypertension up to one year after childbirth. Interestingly, preterm delivery was a protective factor, suggesting that a shorter exposure to pregnancy protected kidney function.

Hypertension was the most common cause of kidney failure in our population. Regarding reproductive history, hypertension was the most common condition before, during, and one year post-pregnancy, and preeclampsia was associated with ESRD. However, in many young women with arterial hypertension, ESRD can be attributed to hypertensive nephrosclerosis, which is not always the reality, as there are cases where undiagnosed glomerulonephritis can be the primary cause of ESRD^8,9^. Our results suggest an association between preeclampsia and APO and hemodialysis, which is in line with evidence published elsewhere. A systematic review with meta-analysis showed that the odds ratio of developing CKD and ESRD with a history of preeclampsia was 2.11 (95%CI 1.72–2.59) and 4.90 (95%CI 3.56–6.74), respectively^10^. Another study found a 4.7 (95%CI 3.6–6.1) relative risk of developing ESRD in women who have had preeclampsia during pregnancy^11^. One cohort study from Sweden showed that hypertension disorders during pregnancy, with an emphasis on preeclampsia, are associated with later CKD, especially if the identified cause of CKD was hypertension or diabetes^12^.

Preeclampsia is the leading cause of acute kidney injury during pregnancy and may be linked to pre-existing or non-diagnosed CKD^3^. It is essential to realize that the effects of disease or exposure during pregnancy persist throughout women’s reproductive cycles^13^. We also emphasize the importance of contraceptive guidance after a pregnancy with an unfavorable outcome to investigate possible underlying diseases and avoid putting a new burden on the organs and systems with a new pregnancy before fully knowing the risks.

A study from Norway linked a repeated history of preeclampsia history with a higher relative risk of ESRD. Women with one pregnancy and a history of preeclampsia increased their risk of ESRD by 3.2%. In contrast, those with two or more pregnancies with preeclampsia increased their risk to 15.5%^11^. The majority of women in our sample had three or more pregnancies, with a high prevalence of hypertension and preeclampsia. The repeated pregnancies probably increased the risk of developing CKD. Early diagnosis of CKD was difficult due to limited access to the health system, which affected early onset of treatments to avoid or delay ESRD.

It is difficult to determine whether preeclampsia causes later ESRD with the influence of genetic and environmental factors or if the real physiopathology is that women with subclinical and undiagnosed kidney injury are prone to developing preeclampsia during pregnancy. Unfortunately, in most pregnant women who start prenatal care with a history of hypertension, there is no previous diagnosis with biopsy or renal function study. Especially in younger women with no other risk factors for essential hypertension, there must be an underlying cause for the early onset of hypertension. Among the causes of secondary hypertension in young women, glomerular disease (eg, lupus nephritis, IgA nephropathy) is the cause rather than the consequence of high blood pressure^14^. Nonetheless, this study indicates the importance of completing early renal function screening in hypertensive women with a history of preeclampsia to improve early diagnosis and slow the development of ESRD. Studies have shown that primary CKD screening is cost-effective^15,16^.

The fact that most included women had low educational levels, low household incomes, and non-white ethnicity reflects the reality in Brazil, where poverty may hamper access to health systems. Our findings agree with the Global Burden of Disease study^1^, which showed that most cases and deaths from CKD occur in populations with low, low-middle, and middle socio-demographic index. A person with CKD on hemodialysis indicates final stage of a disease that could be controlled or postponed if the cause were addressed or treatment initiated early. Deficiencies in health systems and the particular vulnerability of patients with kidney failure must be anticipated and addressed prospectively^12^. Understanding that women with APO and preeclampsia or persistent hypertension are at high risk for CKD reinforces the need for adequate follow-up of kidney function before ESRD onset.

Interestingly, more than half of our population had their first pregnancy before 20 years of age. Vulnerability and low access to health systems result in lower family planning, which leads to unplanned pregnancies. These women will be unnecessarily exposed to complications such as hypertension and preeclampsia, probably increasing their risk of developing CKD.

Our study has some strengths. We interviewed 237 women undergoing hemodialysis, selecting 208 with past pregnancies. Few studies have correlated CKD to reproductive history, and our study filled a knowledge gap regarding the impact of reproductive history on occurrence of ESRD. However, our study had some limitations that should be mentioned. Our data were collected by interviews with women; in two-thirds of them, more than ten years elapsed between the last pregnancy and the renal failure diagnosis. Therefore, some information may have been forgotten or confounded, and there were some missing data, particularly regarding renal disease status. In addition, the lack of knowledge of hemodialysis patients about their disease is an issue that needs to be valued and addressed by the health teams that treat these patients^17^. In women with more than one pregnancy, the pregnancy index of adverse outcomes was not identified because of the limitation of the method. However, in obstetric history, the occurrence of any adverse outcome is what matter most. Another limitation was that women were selected in 4 hemodialysis centers in southeastern Brazil, the country’s most prosperous region.

## Conclusions

Hypertension was the most prevalent cause of CKD among women undergoing hemodialysis in a Brazilian center. Among these women, CKD due to hypertension was associated with gestational hypertension or any hypertension during pregnancy, and also with hypertension up to one year after birth. Health services that assist women at any stage of life should be aware of factors in their reproductive history that may lead to risk for kidney disease to improve early referral to appropriate specialists and prevention or postponement of ESRD.

## Supplementary Material

The following online material is available for this article:

Data collection of a prospective study of patients in hemodialysis.
