# Supplementary material for: Gestational hypertension as a factor associated with chronic kidney disease: the importance of obstetric history of women undergoing hemodialysis
Source: J Bras Nefrol. 2023 Jan 9;45(3):294–301. doi: 10.1590/2175-8239-JBN-2022-0119en (PMC10697153; doi:10.1590/2175-8239-JBN-2022-0119en)
Supplement: Supplementary file 1 [file 2175-8239-jbn-2022-0119-s1.pdf]

Braz. J. Nephrol. (J. Bras. Nefrol.)
https://doi.org/10.1590/2175-8239-JBN-2022-0119en

Supplementary Material for "Gestational hypertension as a factor associated with chronic kidney disease: the importance of obstetric history among women undergoing hemodialysis"

DATA COLLECTION OF A PROSPECTIVE STUDY OF PATIENTS IN HEMODIALYSIS (ESRD)

Form No.: DN: __/__/____ Name: ___________________________________

I. Personal information

1. Marital status: ( ) Single ( ) With partner ( ) Widow ( ) Separated

2. Profession: __________________

3. Education: ( ) None ( ) Elementary ( ) Middle ( ) Higher - incomplete ( ) Higher - complete

4. Type of home: ( ) Rural ( ) Urban

5. Color: ( ) White ( ) Yellow ( ) Brown ( ) Black

6. How many residents are responsible for the total household income? _______________

7. Economic level: what is the total household income: ______________________________

8. Smoking: ( ) Yes ( ) No. If yes, how many cigarettes do you smoke per day?___________

9. Alcoholism: ( ) Yes ( ) No. If yes, how much per day? _______________________

10. Drugs: ( ) Yes ( ) No. If yes, what type and frequency of use:____________________

11. HIV serology: ( ) Yes ( ) No

12. Hep C serology: ( ) Yes ( ) No

13. Hep B serology: ( ) Yes ( ) No

II. Information about the disease

1. Cause of the disease: ( ) AH ( ) DM ( ) UTI ( ) Autoimmune ( ) other __________ ( ) Don't know

2. Time on hemodialysis treatment (in years):_____________________________

III. Information about pregnancies

1. Pregnancies (if pregnant, include current pregnancy):____ Vaginal deliveries:____ Cesarean deliveries:____ Miscarriage:____ Number of living children:____

2. Age at first pregnancy (in years):______

3. Age at last pregnancy (in years):_____________________

4. Interval between last pregnancy and disease diagnosis (in years): _______

5. Pregnancy after disease diagnosis: ( ) Yes ( ) No

6. Pathological condition before pregnancy(ies):

    A. Diabetes: ( ) Yes ( ) No

    B. Hypertension: ( ) Yes ( ) No

    c. Heart disease: ( ) Yes ( ) No

    d. Urinary tract infection: ( ) Yes ( ) No

    e. Lupus: ( ) Yes ( ) No

    f. Others:______________________

7. Pathological condition during pregnancy:

a. Diabetes during pregnancy: ( ) Yes ( ) No

b. Chronic hypertension: ( ) Yes ( ) No

c. Preeclampsia: ( ) Yes ( ) No

d. Eclampsia: ( ) Yes ( ) No

e. Bleeding during pregnancy, childbirth, or puerperium: ( ) Yes ( ) No

f. Preterm labor: ( ) Yes ( ) No

g. Placental abruption: ( ) Yes ( ) No

h. Others:___________________________

8. Pathological condition after pregnancy (diagnosis up to one year after the gestational outcome):

a Diabetes: ( ) Yes ( ) No

b. Hypertension: ( ) Yes ( ) No

c. Heart disease: ( ) Yes ( ) No

d. Urinary tract infection: ( ) Yes ( ) No

e. Lupus: ( ) Yes ( ) No

f. Others:________________________

18. Unfavorable perinatal outcomes:

a. Abortion : ( ) Yes ( ) No

b. Fetal death: ( ) Yes ( ) No

c. Neonatal death: ( ) Yes ( ) No

d. Premature birth: ( ) Yes ( ) No

e. Low birth weight: ( ) Yes ( ) No

f. Others:_________________________

Is there any special situation related to pregnancy and kidney disease that you remember and would like to report?

____

____

____

____

____

____
